# Loneliness and parental caregiving burden in families of children with Prader-Willi syndrome in China: the moderating effects of family functioning and socioeconomic status

**DOI:** 10.3389/fpsyg.2026.1771064

**Published:** 2026-05-29

**Authors:** Nueraili Dayimu, Lingli Leng, Gulisitan Kuerbanniyazi, Xiaojing Lin

**Affiliations:** 1 Department of Sociology, Zhejiang University, Hangzhou, China; 2 School of Statistics and Data Science, Xinjiang University of Finance and Economics, Urumqi, China; 3 Prader-Willi Syndrome Care and Support Center of China, Hangzhou, China

**Keywords:** caregiving burden, family function, family SES, loneliness, Prader-Willi syndrome, primary caregiver, rare disease

## Abstract

**Background:**

Raising a child with Prader-Willi syndrome (PWS) is associated with substantial caregiving challenges for parents. Elevated levels of caregiving burden and loneliness have been documented in this population. However, the relationship between loneliness and caregiving burden, as well as the factors that may shape this association, remains insufficiently understood.

**Objectives:**

This study aimed to examine the association between loneliness and parental caregiving burden, particularly regarding the moderating role of family functioning and family SES.

**Methods:**

A cross-sectional survey was conducted among 1,105 children with PWS and their parent caregivers through *the Prader-Willi Syndrome Care and Support Center* from July to October, 2023. Participants completed a questionnaire assessing sociodemographic factors, self-rated health (EQ-5D-5L), caregiving burden (ZBI-22), loneliness (De Jong Gierveld-11), and family function (Apgar-5). Descriptive statistics and hierarchical multiple linear regression analyses were conducted in R 4.2.2. We employed a nonparametric bootstrapping for sensitivity analysis.

**Results:**

Results indicated: (1) Loneliness was positively associated with caregiving burden. (2) Family SES was negatively associated with caregiving burden. (3) Family functioning negatively moderates the association between loneliness and caregiving burden. (4) Family SES positively moderates the association between loneliness and caregiving burden.

**Conclusion:**

This study identifies that loneliness was positively associated with caregiving burden among parents of children with PWS in China. Family functioning buffers the effect, whereas higher SES may amplify it. These findings highlight the importance of promoting family cohesion, financial stability, PWS-specific social welfare, and reducing parental loneliness.

## 1 Introduction

Prader-Willi syndrome (PWS) is a rare neurodevelopmental genetic disorder, which affects approximately 1 in 15,000 to 30,000 individuals worldwide (Butler et al., 2019; Duis et al., 2019). PWS is characterized by severe hypotonia with poor suck and feeding difficulties in infancy, followed by excessive hyperphagia and marked obesity in early childhood, along with serious emotional and behavioral problems such as tantrums, compulsions, and cognitive impairments (Butler et al., 2019; Cassidy et al., 2012; Duis et al., 2019). Research has shown that the predominant symptoms of PWS, along with the associated behavioral challenges, contribute to higher levels of perceived caregiver burden (Amaro et al., 2022; Bos-Roubos et al., 2022; Chiu et al., 2017; Lanfranchi and Vianello, 2012; Maccarone et al., 2024; Rozensztrauch and Śmigiel, 2022; Kayadjanian et al., 2021; Mackay et al., 2019; Mackay et al., 2022). In addition, limited medical resources, diagnostic delays, and the complexity of disease management often magnify caregiving burden among parents of children with PWS (Atkins and Padgett, 2024; Tvrdik et al., 2015; Kowal et al., 2022; Mackay et al., 2022; Usubini et al., 2024; Boettcher et al., 2021; Huang et al., 2020; Mao et al., 2019; Mora-Lagunes Recinos et al., 2025).

Perceived caregiving burden has been defined as the perception of stress and burden, which is subjectively assessed by those who provide long-term care (Zarit et al., 1980). Prior literature have documented the positive association between caregiving burden and adverse mental health outcomes, including depression, anxiety, emotional distress, and suicidal ideation (Hu et al., 2018; Clyburn et al., 2000; Solimando et al., 2022; Sun et al., 2019; González Ruiz et al., 2024; Micallef Pulè and Hughes, 2025). Moreover, existing research demonstrated higher prevalence of perceived caregiving burden among the primary caregivers of children with PWS (Kayadjanian et al., 2018; Kayadjanian et al., 2021; Chiarotti et al., 2023; Usubini et al., 2024; Walkowiak and Domaradzki, 2025a, 2025c; Walkowiak and Domaradzki, 2025b; Walkowiak et al., 2025). Those findings underscore the critical need to address caregiving burden and its contributing factors to improve overall well-being of primary caregivers within PWS. However, there is insufficient knowledge about factors associating with perceived caregiving burden and its conditions within this population, especially in mainland China.

### 1.1 Loneliness and perceived caregiving burden

Previous studies have found that loneliness predicts caregiver burden longitudinally within the spousal caregivers of Alzheimer’s disease (Fagundes et al., 2025). Studies also have found positive association between loneliness and caregiving burden within pediatric care (Kobos et al., 2023). In addition, Chow et al. (2025) found a positive relationship between loneliness and caregiving burden among parents of children with rare Down Syndrome Regression Disorder. Loneliness was defined as an individual’s subjective assessment of social isolation and a lack of social connections (De Jong Gierveld and Van Tilburg, 2010). Prior research has shown that having a family member with a rare disease negatively impact caregivers’ social relationships (Atkins and Padgett, 2024; Silibello et al., 2016; Pelentsov et al., 2016), often leads increased loneliness (Sandilands et al., 2022). Studies on PWS have also highlighted severe loneliness among caregivers (Filangieri and Singh, 2022; Mazaheri et al., 2013; Currie et al., 2024a,b). For example, Kowal et al. (2022), through qualitative interviews, found that a lack of understanding from the broader social environment regarding the disease and its care needs exacerbated feelings of loneliness among parents of children with PWS. However, research on the relationship between loneliness and caregiving burden within the context of PWS remains limited. We hypothesize:

Hypothesis 1: Loneliness may positively associate with perceived caregiving burden among the parent caregivers of children with PWS in China.

### 1.2 Family functioning and perceived caregiving burden

Family functioning defined as a commitment of relationship for members to nurture each other when family equilibrium is stressed during a crisis (Smilkstein, 1978). It is defined as family member’s ability to maintain cohesive relationships with one another to cope with family problems (Zhang, 2018). Research have found positive correlation with family functioning and health related quality of life in all dimensions among family caregivers of dependent people in Spain (Rodríguez-Sánchez et al., 2011). Ribé et al. (2018) also found a negative association between family functioning and caregiving burden among the family caregivers of patients with schizophrenia in Spain. In addition, recent research has shown that family functioning significantly moderates the negative association between psychological distress and quality of life among caregivers of patients with advanced cancer in China (Cui et al., 2024). Thus, we hypothesize:

Hypothesis 2a: Family functioning may negatively correlate with perceived caregiving burden among the parent caregivers of children with PWS in China.

Hypothesis 2b: Higher family functioning may negatively moderate the relationship between loneliness and caregiving burden among the parents of children with PWS in China.

### 1.3 Family SES and perceived caregiving burden

Fundamental cause theory posits that socioeconomic status (SES) is a key determinant of health inequalities, as it shapes access to and utilization of medical resources (Phelan et al., 2010). Consistent with this perspective, prior research has documented socioeconomic disparities in perceived caregiving burden among caregivers of older patients with cancer in China (Ju et al., 2025). However, objective socioeconomic advantage does not necessarily translate into better subjective well-being. Relative deprivation theory suggests that individuals evaluate their circumstances through social comparisons and may feel deprived when they perceive themselves as worse off than relevant others (Walker and Pettigrew, 1984). Notably, even individuals with higher SES may experience relative deprivation when comparing themselves with similarly advantaged peers within their social reference group (Callan et al., 2015, 2017).

In the Chinese context, influenced by Confucianism, children’s outcomes are closely tied to parental “face,” and parents’ sense of self-worth is often contingent on children’s fulfillment of social expectations (Leng et al., 2024; Ng et al., 2014). Higher SES families are typically embedded in networks of similarly advantaged peers, where expectations for children’s achievement are particularly salient (Davis-Kean, 2005; Poon, 2020). Consequently, having a child with a rare disease may be perceived as a threat to face, which may lead parents to feel ashamed and withdraw from social networks in China (Dayimu et al., 2026; Xu, 2023). Within this context, parents of children with PWS from higher SES families may be particularly vulnerable to relative deprivation, as their child’s outcomes fall short of culturally reinforced expectations of success.

In addition, limited availability of specialized healthcare resources has been shown to hinder service utilization even among higher SES families in China (Ye et al., 2019), which may further intensify perceived disadvantage when adequate resources for PWS are scarce (Dempsey et al., 2025). Therefore, we hypothesize:

Hypothesis 3a: Based on fundamental cause theory, family SES may negatively associate with perceived caregiving burden within PWS in China.

Hypothesis 3b: Based on relative deprivation theory, family SES may positively moderate the association between loneliness and caregiving burden within PWS in China.

## 2 Data and methods

### 2.1 Participants

This study employed a cross-sectional design and conducted an online survey from July to October, 2023. We defined the primary caregiver is the person who provides the most consistent and substantial care for the patient on a daily basis, typically one of the parents in our study design. In cases where multiple caregivers are involved, we will include only the person who provides the majority of care and who assumes the primary caregiving responsibilities. The inclusion criteria for this study require participants to meet the following conditions simultaneously: (1) The participant must be the parent of the patient; (2) The participant must be the primary caregiver as defined above; (3) The patient must be clinically diagnosed with PWS; (4) The participant must be cognitively functional and able to independently participate in the study (i.e., able to provide informed consent and complete necessary assessments); (5) The participant must be able to read and write; (6) The participant must be a resident of mainland China and currently living there. The exclusion criteria for this study specify that participants will be excluded if they meet any one of the following conditions: (1) Non-parent caregivers, such as grandparents, relatives, or other legal guardians; (2) Caregivers with cognitive impairment or severe mental health conditions that would impair their ability to participate meaningfully in the study; (3) Caregivers who have difficulty understanding the study content; (4) Caregivers who are unable or unwilling to provide informed consent; (5) Caregivers who are not residents or are not currently living in mainland.

Due to the absence of a definitive epidemiological survey and the difficulties in accessing this specific population, we utilized convenience sampling to recruit participants through the Prader-Willi Syndrome Care and Support Center, which is the only national umbrella organization for patients with PWS in mainland China. This organization was established in 2017 by family members of PWS patients to support individuals with PWS and their families through education, medical rehabilitation, psychological support, employment support, and corresponding policy advocacy. Currently, over 2,000 individuals with PWS are registered with this organization. We collected a total of 1,105 valid questionnaires for children aged less than 18. Each questionnaire represents one child with PWS and one primary caregiver among their parents. The basic characteristics of participants were summarized in Table 1. Approximately two thirds of caregivers were mothers, most of them live in cities, and more than half located in the developed eastern regions of China. Among the children with PWS, majority were girls and older than 6 years. Most of the children were diagnosed 3.7 ± 3 years before the survey.

**Table 1:** Participant characteristics.

Primary caregiver’s characteristics			Children’s characteristics		
Variables	Mean (*N*)	SD (%)	Variables	Mean (*N*)	SD (%)
Primary caregiver			Sex		
Father	409	37.0%	Boys	437	39.5%
Mother	696	63.0%	Girls	668	60.5%
Self-rate physical health	2.20	1.31	Age by interval, years		
Self-rate mental health	2.77	1.22	≤3.0	218	19.7%
Multiple caregivers			3.1–6.0	220	19.9%
Yes	611	55.3%	6.1–12.0	479	43.3%
No	494	44.7%	12.1–18.0	188	17.0%
Place of residence			Time since diagnosis, years	3.6	2.9
Rural	497	45.0%	Complication		
Urban	608	55.0%	Yes	106	9.6%
Region			No	999	90.4%
East China	572	51.8%	Intellectual disability		
Middle China	355	32.1%	Yes	916	82.9%
West China	178	16.1%	No	189	17.1%
			Disability certificate		
			Yes	460	41.6%
			No	645	58.4%

SD is standard deviation. N is Frequency. % is Percentage.

All data were collected anonymously, in compliance with the requirements of the Statistics Law of the People’s Republic of China as well as the Declaration of Helsinki. The study protocol was approved by the Institutional Review Board of Children’s Hospital, Zhejiang University School of Medicine (Ethics ID: 2024-IRB-0152-P-01). Written informed consent was acquired from all participants after clarifying the purposes of the study.

### 2.2 Measurement

#### 2.2.1 Perceived caregiving burden

We used the 22-item version of the Zarit Burden Interview (ZBI-22) to assess the perceived caregiving burden of primary caregivers (Zarit et al., 1980). The scale consists of 22 items and employs a 5-point Likert scale assessment. Participants rated each item based on their personal feelings, with scores ranging from 0 (never) to 4 (always). The total score of the scale ranges from 0 to 88, with higher scores indicating a severe degree of perceived caregiving burden. The Chinese version of this scale exhibited good reliability and construct validity in previous research (Lu et al., 2009; Liang et al., 2025). In this study, the Cronbach’s *α* coefficient for the scale was 0.93, demonstrating excellent internal consistency and reliability.

#### 2.2.2 Loneliness

We administered the De Jong Giervel Loneliness scale to evaluate primary caregiver’s feelings of loneliness. This scale contains 11 items and applies a 5-point Likert scale. Participants assess each item based on their subjective feelings from “never of the time” to “All of the time” (De Jong Gierveld and Van Tilburg, 2010). The Chinese version of this scale demonstrated good reliability and construct validity in prior research (Cheung et al., 2022). The total score of the scale ranges from 0 to 11, with higher scores suggesting an increased level of loneliness. In this study, the Cronbach’s α coefficient for the scale was 0.85, proving good internal consistency and reliability.

#### 2.2.3 Family SES

Family SES was measured using a composite index that including annual household income, parents’ education (both mother’s and father’s), and parents’ employment status as reported by participants (Gao, 2022; Vyas and Kumaranayake, 2006; Wu et al., 2018). First, participants reported the highest educational level that they attained by selecting one of six options: primary school, secondary school, high school, community college, university, and graduate or higher, with codes ranging from 1 to 6. Annual household income level was categorized into five groups: <50,000, 50,000–100,000, 100,000–200,000, 200,000–500,000, and ≥500,000, with each category coded from 1 to 5. Parents’ employment status was coded as 1 = both parents were unemployed, 2 = only one parent was employed, 3 = both parents were employed.

**Figure 1:**
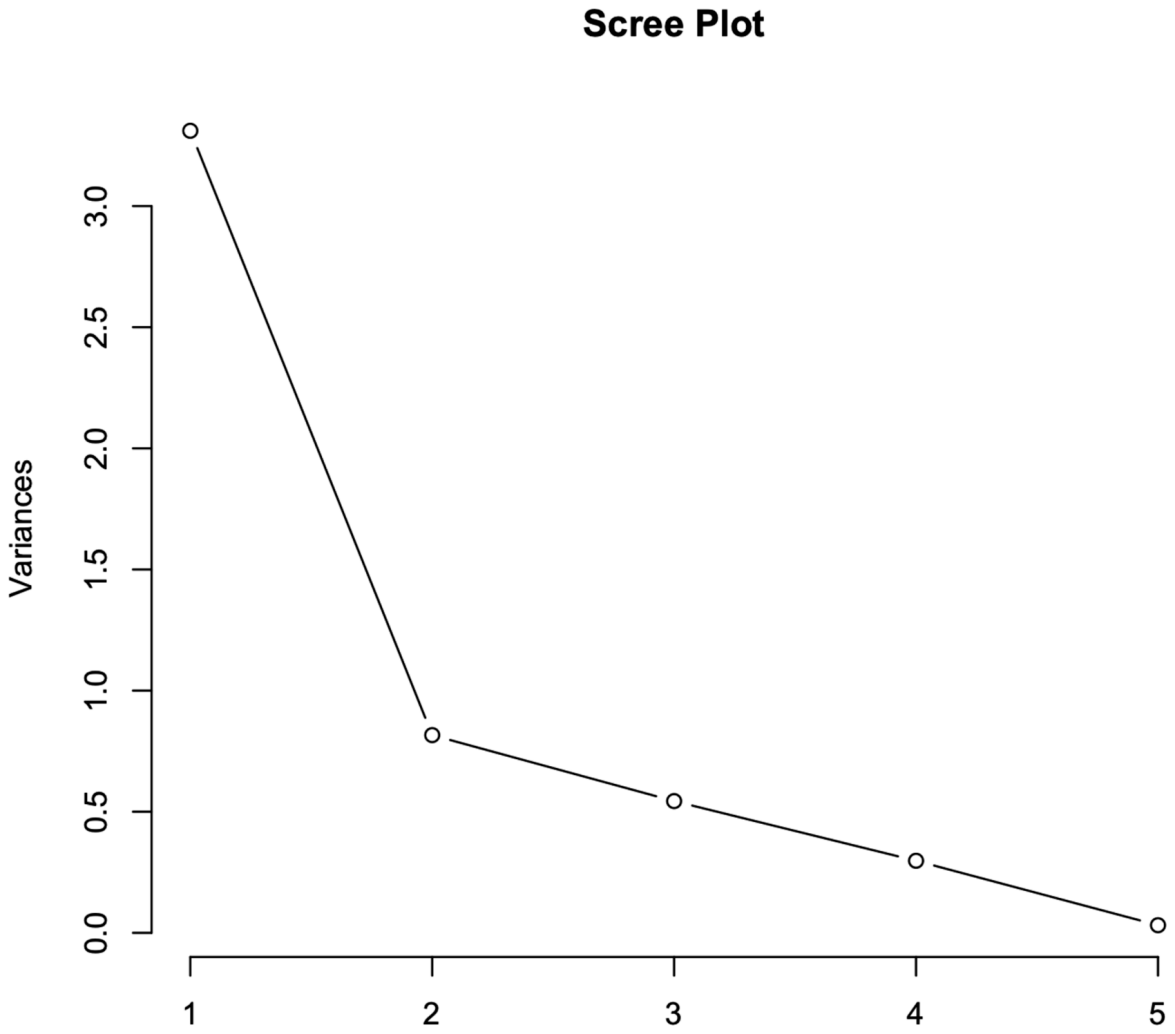
Scree plot of eigenvalues from principal component analysis of parental education, employment status, and household income.

To construct a unified SES variable, Principal Component Analysis (PCA) was used to synthesize these categorical measures into a single score representing family SES (Jolliffe and Cadima, 2016). PC1 explains 58.7% of total variance, and there is clear drop from PC1 to PC2, and eigenvalue of PC1 > 1, thus we retained PC1 as a composite SES index. Figure 1 and Table 2 present the eigenvalues, proportion of variance explained, and component loadings from the Principal Component Analysis of family SES indicators.

**Table 2:** Principal component analysis results for constructing family SES.

**(A)** Component loadings	PC1	PC2	PC3	PC4
Household income	0.487	−0.336	−0.800	0.098
Father education	0.541	0.443	0.057	−0.714
Mother education	0.540	0.419	0.237	0.690
Employment	0.422	−0.718	0.548	−0.082

(B) Component statistics	PC1	PC2	PC3	PC4
Eigenvalue	2.347	0.810	0.545	0.299
Proportion of variance	0.587	0.203	0.136	0.075
Cumulative proportion	0.587	0.789	0.925	1.000

#### 2.2.4 Family functioning

We used the APGAR scale to assess primary caregiver’s feelings of family functioning. The scale contains 5 items: adaptation, partnership, growth, affection and resolve, and applies a 3-point Likert scale (Smilkstein, 1978). Participants assess each item based on their subjective feelings from (hardly ever = 0) to (almost always = 2). The total score ranges from 0 to 10, with higher scores indicating higher levels of family functioning. The Chinese version of APGAR has been widely applied in China with excellent validity and reliability (Huang et al., 2021). In this research, Cronbach’s *α* of the scale was 0.86, showing good validity.

#### 2.2.5 Covariates

Based on prior literature (Bull et al., 2025; Cardinali et al., 2019; Chen et al., 2022; Dempsey et al., 2025; Kayadjanian et al., 2018, 2021; Kowal et al., 2022; Meade et al., 2021; Ihara et al., 2014; Kim et al., 2010), this study included a series of variables as covariates in our analysis to account for potential confounding factors, including:

##### 2.2.5.1 Child-level

Sex (Boys = 0), age (Due to differences of child symptoms, care demands, and parent worries across infancy, childhood, and adolescence within PWS syndrome, child age was categorized into four groups: ≤ 3 years, 3.1–6 years, 6.1–12 years, and 12.1–18 years; ≤ 3 years = 0), time since diagnosis, complication (Yes = 0), intellectual disability (No = 0), disability certificate (No = 0). Complication was asked by “Whether your child with PWS having any other severe diseases?”; intellectual disability was asked by “Whether your child with PWS having lower IQ levels than their peers?”; disability certificate is issued by *the China Disability Union*, having a disability certificate indicates severe disability in China, we get this information asking the parents by “Whether your child with PWS having a disability certificate?”

##### 2.2.5.2 Caregiver-level

Caregiver (Father = 0), self-rated physical health (Measured by EQ-5D-5L scale-mobility), self-rated mental health (Measured by EQ-5D-5L scale-depression), multiple caregivers (No = 0). In addition, to account for structural disparities in healthcare access, economic development, and sociocultural contexts (Lei et al., 2019), we included residence (Urban versus Rural = 0) and geographic region (East, Middle, and West China = 0) as covariates.

### 2.3 Statistical analysis

All analyses were conducted using R version 4.4.2. First, descriptive statistics summarized sample characteristics, with categorical variables presented as n (%) and continuous variables as mean (SD). A principal component analysis was performed to construct a composite family SES using household income, father’s education, mother’s education, and parental employment status.

Second, correlation between caregiving burden, loneliness, family SES, and family functioning were examined using Pearson’s correlation, after evaluating skewness and kurtosis. Loneliness and family functioning were mean-centered using the ‘scale’ function prior to regression analysis.

Third, we conducted hierarchical multiple linear regression to examine the association between loneliness and caregiving burden and the moderating roles of family SES and family functioning. Model 1 included covariates; Model 2 added loneliness; Model 3 further included family SES and family functioning; Model 4 added the interaction terms between loneliness and the moderators. ANOVA was used to test differences in model variance. To ensure robustness, nonparametric bootstrap sensitivity analyses (5,000 iterations) were performed for Model 2–4, with 95% bias-corrected confidence intervals.

Finally, Johnson-Neyman simple slope analyses examined the effect of loneliness on caregiving burden across different levels of family functioning and family SES. Statistical significance was set at *p* < 0.05 (*α* = 0.05) with the bootstrap seed fixed at 123. All significance tests were two-tailed.

All models showed no evidence of problematic multicollinearity (VIF < 5; see Supplementary Table S1). Model assumptions—including linearity, normality, and homoscedasticity—were evaluated using diagnostic plots, which are provided in Supplementary Figures S1–S4.

## 3 Results

### 3.1 Levels of perceived caregiving burden, loneliness, and family function

The average score of perceived caregiving burden was 55.25 ± 17.09 (Median = 55.0, Q1 = 44.0, Q3 = 68.0), skewness was −0.13, kurtosis was −0.53. The average loneliness score was 7.29 ± 2.29 (Median = 8.0, Q1 = 6.0, Q3 = 10.0), skewness was −0.57, kurtosis was −0.18. The average score of family function was 6.11 ± 2.66 (Median = 6.0, Q1 = 5.0, Q3 = 8.0), skewness was −0.23, kurtosis was −0.58. Skewness and kurtosis of all variables indicate approximate normal distribution. Pearson’s correlation results indicate that there was a small-to-moderate positive correlation between loneliness and caregiving burden (*r* = 0.26, *p* < 0.001), small-to moderate negative correlation between family SES and caregiving burden (*r* = −0.22, *p* < 0.001), negative trivial correlation between family functioning and caregiving burden (*r* = −0.06, *p* < 0.05), moderate negative correlation between family functioning and loneliness (*r* = −0.31, *p* < 0.001), small-to-moderate negative correlation between family SES and loneliness, and small-to-moderate negative correlation between family functioning and family SES (*r* = 0.14, *p* < 0.001) (see Table 3).

**Table 3:** Descriptive statistics and Pearson’s correlation matrix of the study variables.

Variable	Mean	SD	Skewness	Kurtosis	1	2	3	4
1. Caregiving burden	55.25	17.09	−0.13	−0.53	1.00			
2. Loneliness	7.92	2.29	−0.57	−0.18	0.26***	1.00		
3. Family function	6.11	2.66	−0.23	−0.58	−0.06*	−0.31***		
4. Family SES	0	1.53	0.08	−0.8	−0.22***	−0.16***	0.14***	1.00

SD is standard error. ^+^*p* < 0.10, ^*^p < 0.05, ^**^p < 0.01, ^***^p < 0.001.

### 3.2 Hierarchical multiple linear regression analysis

Table 4 presents the results of the hierarchical regression analyses, while Table 5 reports the corresponding nonparametric bootstrapped sensitivity analyses of the regression models. In Model 2, the focal predictor loneliness was significantly associated with caregiver burden (*β* = 1.02, 95% bootstrapped CI [0.661, 1.384], *p* < 0.001), indicating that loneliness remained a positive association with caregiving burden after controlling for all covariates (Hypothesis 1).

**Table 4:** Hierarchical regression models.

Predictors	Model 1				Model 2				Model 3				Model 4 (Moderation Model)			
	*Coeff.*	*SE*	*t*	*p*	*Coeff.*	*SE*	*t*	*p*	*Coeff.*	*SE*	*t*	*p*	*Coeff.*	*SE*	*t*	*p*
(Intercept)	33.48	2.57	13.05	**<0.001**	33.70	2.53	13.32	**<0.001**	33.24	2.55	13.05	**<0.001**	33.65	2.55	13.20	**<0.001**
Caregiver (Father = 0)	2.71	0.92	2.96	**0.003**	2.57	0.91	2.84	**0.005**	2.58	0.90	2.85	**0.004**	2.37	0.91	2.61	**0.009**
Self-rated physical health	1.96	0.39	5.08	**<0.001**	2.10	0.38	5.52	**<0.001**	1.94	0.38	5.05	**<0.001**	1.98	0.38	5.16	**<0.001**
Self-rated mental health	6.14	0.41	15.14	**<0.001**	5.71	0.41	14.03	**<0.001**	5.75	0.41	14.15	**<0.001**	5.63	0.41	13.79	**<0.001**
Multiple caregivers (No = 0)	−5.17	0.85	−6.10	**<0.001**	−4.56	0.84	−5.41	**<0.001**	−4.03	0.88	−4.57	**<0.001**	−4.07	0.88	−4.63	**<0.001**
Residency (Rural = 0)	−0.45	0.83	−0.54	0.588	−0.20	0.82	−0.24	0.808	0.53	0.90	0.59	0.557	0.47	0.90	0.52	0.601
Region (West China = 0)																
East China	1.73	1.16	1.49	0.136	1.81	1.15	1.58	0.114	1.96	1.15	1.71	0.087	1.93	1.14	1.69	0.092
Middle China	1.37	1.25	1.09	0.275	1.37	1.23	1.11	0.266	1.29	1.23	1.05	0.295	1.27	1.23	1.04	0.300
Child sex (Boy = 0)	−0.69	0.87	−0.79	0.429	−0.71	0.86	−0.83	0.409	−0.79	0.85	−0.93	0.353	−0.77	0.85	−0.91	0.365
Child age (≤3 years = 0)																
3.1–6 years	−2.32	1.34	−1.72	0.085	−2.31	1.33	−1.74	0.082	−1.98	1.33	−1.49	0.137	−2.00	1.32	−1.51	0.131
6.1–12 years	−4.27	1.25	−3.41	**0.001**	−3.79	1.24	−3.07	**0.002**	−3.57	1.24	−2.89	**0.004**	−3.46	1.23	−2.81	**0.005**
12.1–18 years	−6.08	1.56	−3.90	**<0.001**	−5.69	1.54	−3.69	**<0.001**	−5.58	1.54	−3.63	**<0.001**	−5.55	1.53	−3.62	**<0.001**
Time since diagnosis	0.88	0.16	5.33	**<0.001**	0.83	0.16	5.08	**<0.001**	0.82	0.16	5.06	**<0.001**	0.82	0.16	5.10	**<0.001**
Complication (Yes = 0)	−3.57	1.42	−2.51	**0.012**	−3.50	1.40	−2.50	**0.013**	−3.58	1.41	−2.55	**0.011**	−3.84	1.40	−2.73	**0.006**
IQ deficiency (No = 0)	3.91	1.12	3.48	**0.001**	3.96	1.11	3.57	**<0.001**	3.95	1.11	3.57	**<0.001**	4.02	1.10	3.64	**<0.001**
Certificate (No = 0)	2.70	0.89	3.05	**0.002**	2.79	0.87	3.19	**0.001**	2.60	0.88	2.97	**0.003**	2.59	0.87	2.97	**0.003**
Loneliness					1.02	0.18	5.60	**<0.001**	1.09	0.19	5.73	**<0.001**	1.13	0.20	5.73	**<0.001**
Family SES									−0.70	0.32	−2.21	**0.027**	−0.71	0.32	−2.25	**0.025**
Family function									0.31	0.16	1.93	0.054	0.30	0.16	1.90	0.058
Loneliness × SES													0.24	0.11	2.17	**0.030**
Loneliness × Family function													−0.15	0.07	−2.14	**0.033**
Observations	1,105				1,105				1,105				1,105			
*R*^2^/*R*^2^ adjusted	0.388/0.380				0.405/0.397				0.410/0.400				0.414/0.403			
*F*	46.086				46.369				41.889				38.299			
Log. Lik.	−4432.505				−4416.812				−4412.756				−4408.746			
AIC	8899.010				8869.624				8865.513				8861.491			
ΔR^2^	-				0.017***				0.005*				0.004*			

Coeff. is regression coefficients. SE is standard error. *t* is *t*-statistic. *p* is *p*-value. *R*^2^ is r-squared. *F* is F-statistic of overall model. Log.Lik. is Log-likelihood. AIC is Akaike information criterion. ΔR^2^ is the change in explained variance. ^+^ *P* < 0.10, ^*^ *P* < 0.05, ^**^ *P* < 0.01, ^***^ *P* < 0.001. Model 1 is controls only; Model 2 is the main effect of loneliness; Model 3 is the main effects of loneliness, family SES, and family function; Model 4 is interaction model. Bold values indicate statistical significance in the regression model.

**Table 5:** Bootstrap sensitivity analysis of regression models.

Variables	Model 2		Model 3		Model 4 (Moderation)	
	Coeff.	95% CI	Coeff.	95% CI	Coeff.	95% CI
Loneliness	1.021 (0.182)	0.661, 1.384	1.090 (0.190)	0.715, 1.462	1.133 (0.198)	0.752, 1.525
SES			−0.699 (0.317)	−1.316, −0.091	−0.711 (0.316)	−1.327, −0.114
Functioning			0.310 (0.160)	−0.001, 0.624	0.304 (0.161)	−0.001, 0.614
Loneliness × SES					0.244 (0.113)	0.030, 0.465
Loneliness × Family function					−0.149 (0.070)	−0.304, −0.011
Covariates	Controlled		Controlled		Controlled	
Observations	1,105		1,105		1,105	
Bootstrap simulations	5,000		5,000		5,000	

Standard errors in parentheses. Coef. is regression coefficient. CI is bootstrapped confidence interval. Model 2 is the main effect of loneliness; Model 3 is the main effects of loneliness, family SES, and family function; Model 4 is interaction model. Control model (Model 1) is not included to this table. All models are adjusted for caregiver, self-reported physical health, self-reported mental health, multiple caregivers, residency, region, child sex, age intervals, complication, intellectual disability, and disability certificate.

In Model 3, after introducing the moderator variables-family SES and family function, loneliness remained significantly associated with caregiver burden (*β* = 1.09, 95% bootstrapped CI [0.715, 1.462], *p* < 0.001). In addition, family SES showed a significant negative association with caregiving burden (*β* = −0.70, 95% bootstrapped CI [−1.316, −0.091], *p* < 0.05) (Hypothesis 3a). However, the main effect of family function on caregiving burden was not statistically significant (Hypothesis 2a was not proved). In Model 4, which additionally included the interaction terms, loneliness continued to exhibit a significant positive association with caregiving burden (*β* = 1.13, 95% bootstrapped CI [0.752, 1.525], *p* < 0.001), while family SES also remained negatively associated with caregiving burden (*β* = −0.71, 95% bootstrapped CI [−1.327, −0.114], *p* < 0.05).

Model comparison statistics further supported the hierarchical specification. Analysis of variance indicated that F-change in Model 2 (*F* = 31.690, *p* < 0.001), Model 3 (*F* = 4.022, *p* < 0.05), and Model 4 (*F* = 3.949, *p* < 0.05) were all statistically significant. In addition, improvements in model fit were observed across successive models, as indicated by increases in Log-Likelihood and reductions in the Akaike information criterion (AIC), suggesting the later models provided a better fit to the data than the former ones.

### 3.3 Moderation analysis

Model 4 of Tables 4, 5 showed that the production between loneliness and SES was positively significant (*β* = 0.24, 95% bootstrapped CI [0.030, 0.465], *p* < 0.05), indicating that SES positively moderated the association between loneliness and caregiving burden (Hypothesis 3b). In addition, the production between loneliness and family functioning was negatively significant (*β* = −0.15, 95% bootstrapped CI [−0.304, −0.011], *p* < 0.05), indicating that family functioning negatively moderated the association between loneliness and caregiving burden (Hypothesis 2b). However, the Δ*R*^2^ between model 3 and model 4 was 0.004 (*p* < 0.05), indicating the moderation effect was very small.

**Figure 2:**
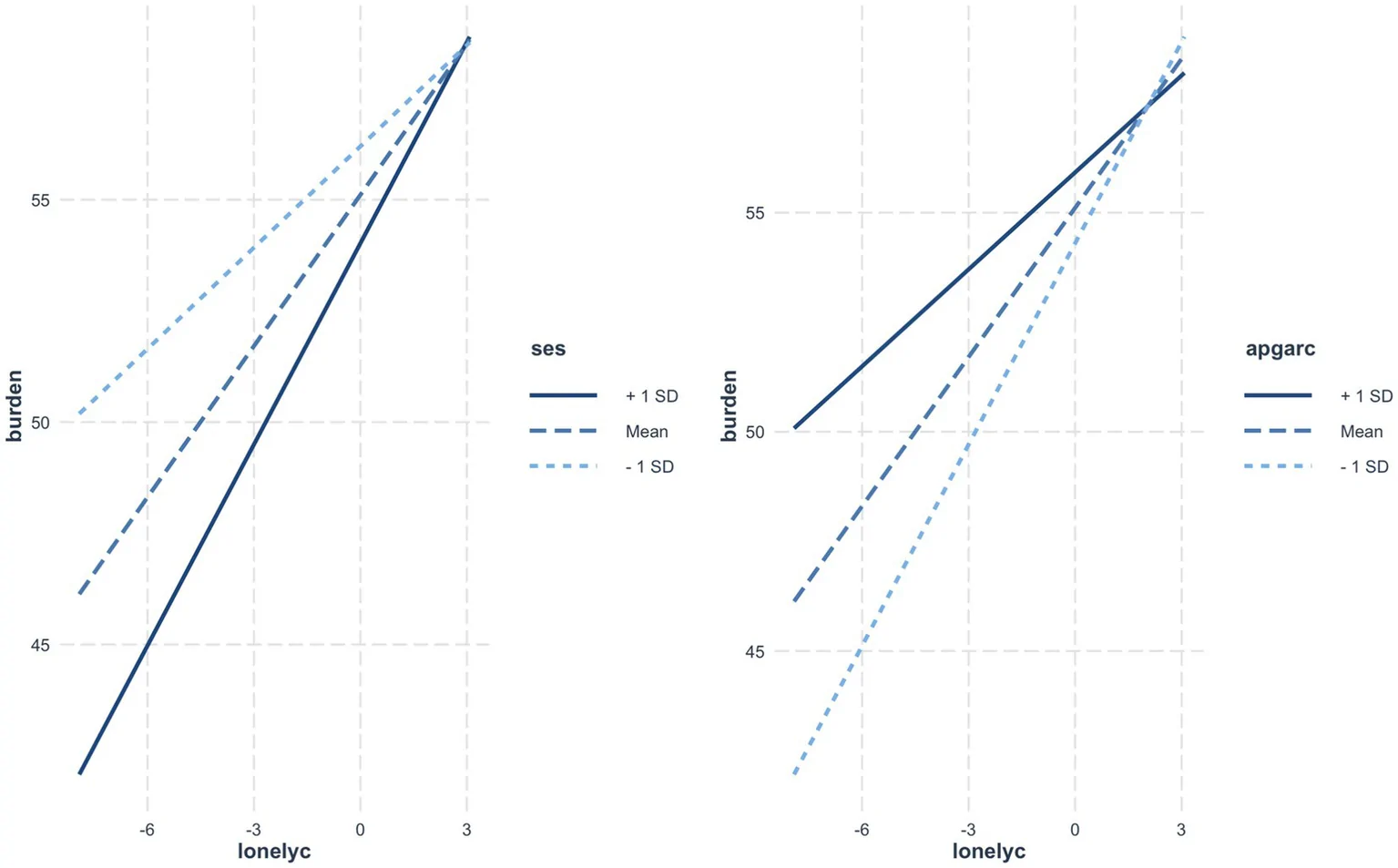
Moderating effects of family SES and family functioning on the relationship between loneliness and caregiving burden.

Johnson-Neyman simple slope analyses revealed that the effect of loneliness on burden was stronger at high SES (*β* = 1.51, *p* < 0.001) than at low SES (*β* = 0.76, *p* < 0.05), while the effect of loneliness on burden was stronger at low family functioning (*β* = 1.53, *p* < 0.001) than at high family functioning (*β* = 0.74, *p* < 0.001). Simple slope analysis results summarized in Table 6 and visualized in Figure 2.

**Table 6:** Simple slopes analysis for the moderating effect of family SES and functionality on the relationship between loneliness and caregiving burden (Johnson-Neyman slope analysis of loneliness).

Family SES	Coeff.	SE	*t*	*p*
Low (− 1 SD)	0.76	0.28	2.74	0.01
Mean (0 SD)	1.13	0.20	5.73	0.00
High (+ 1 SD)	1.51	0.25	6.10	0.00

Family functionality	Coeff.	SE	*t*	*p*
Low (−1 SD)	1.53	0.30	5.08	0.00
Mean (0 SD)	1.13	0.20	5.73	0.00
High (+1 SD)	0.74	0.24	3.11	0.00

Coeff. is slope of loneliness on caregiving burden. SE is standard error. *t* is *t*-statistic. *p* is *p*-value. SD is standard deviation. Low is lower level of moderator. High is higher level of moderator.

## 4 Discussion

Understanding how parents of children with a rare disease subjectively burdened is essential for improving their overall well-being. To our knowledge, this is the first study to examine the association between loneliness and perceived caregiving burden, and the moderating role of family functioning and family SES in this relationship among the parents of children with PWS in mainland China. Our findings reveal that loneliness were positively associated with caregiving burden among the parent caregivers of the children with PWS. Family functioning and family SES played as significant moderators in this relationship. Specifically, family functioning negatively moderated the association between loneliness and parental caregiving burden. Family SES, besides the negatively significant main effects on parental caregiving burden, positively moderates the correlation between loneliness and caregiving burden.

### 4.1 Loneliness and parental caregiving burden

The findings suggest that loneliness is significantly associated with parental caregiving burden. Consistent with prior research, higher levels of loneliness linked with higher levels of caregiving burden (Chow et al., 2025; Fagundes et al., 2025; Kobos et al., 2023). Parents of children with PWS were experiencing higher than average levels of feelings of isolation (Mazaheri et al., 2013). Parents serving as primary caregivers for children with PWS face challenges in maintaining social networks. This difficulty arises from the constant supervision and monitoring of food consumption required by the disease condition, as well as the parent caregiver’s unmet needs for respite care (Tvrdik et al., 2015). Besides, scarcity of existing knowledge (Adama et al., 2023; Crowe et al., 2019; Lyon et al., 2022) makes it difficult for parents as they climb the mountain; they need to navigate the whole journey by themselves alone (Baumbusch et al., 2018; Currie and Szabo, 2019a, 2019b), thus they lack of support—not only professional support (Crowe et al., 2019; Pomey et al., 2015), but also psychological and emotional support (McMullan et al., 2022). Inadequacy of support may associate with severe levels of caregiver burden. Third, high caregiving expenses can contribute to increased loneliness, which may, in turn, exacerbate feelings of being overwhelmed and stressed (Filangieri and Singh, 2022). Fourth, social misunderstandings further compound their struggles. Parents of children with PWS often experience misunderstood by others, including extended family and educational or medical professionals (Cagalj et al., 2018; Dempsey et al., 2025; Schofield et al., 2021; Vitale, 2016), which may also exacerbate caregiving burden.

### 4.2 Socioeconomic disparities of parental caregiving burden

The observed main effect of family SES indicates that parental caregiving burden is socially stratified. Consistent with established health equity research, lower family SES is significantly associated with higher levels of caregiving burden (Ju et al., 2025). One useful lens for interpreting this gradient is the fundamental cause theory of health, which emphasizes that SES shapes the capacity to access and effectively use health-related resources and services (Phelan and Link, 2013). For families having a child with PWS, consistent growth hormone therapy and rehabilitation services are widely recognized as essential for stabilizing children’s conditions (Duis et al., 2019). Yet these interventions are not fully covered by existing social welfare provisions in China (Yu et al., 2024, 2025). families with greater resources are better positioned to secure consistent treatment and navigate complex care systems, whereas lower-SES families may face interruptions in care due to financial constraints. Theses disparities in access and continuity of care likely translate into caregiving demands differences, thereby reinforcing socioeconomic gradients in parental care burden.

### 4.3 The negative moderating role of family functioning

The study found that family functioning negatively moderated the association between loneliness and caregiving burden. In families characterized by stronger cohesion, communication, and role coordination may makes the association between loneliness and caregiving burden weaker. The finding is consistent with family resilience perspectives (Walsh, 2016), which emphasizes the significance of the functioning of the family system in dealing with adversity. In the context of PWS, uncertainty and limited knowledge of the disease often lead to misunderstanding and absence of social support (Dempsey et al., 2025). Supportive family environments may reduce tendencies toward self-isolation and stigma within a rare disease (Dayimu et al., 2026). Better family functioning facilitates family members with mutual relationships, communications, and care (Scully et al., 2020), which may mitigate the adverse mental health outcomes (Wu et al., 2024). Under such conditions, loneliness is less likely to escalate into more generalized sense of burden. This finding highlights the importance of family system within PWS contexts in China. More importantly, it suggests that family functioning operates as a protective factor, buffering the effects of loneliness and isolation on perceived caregiving burden.

### 4.4 The positive moderating role of family SES

The moderation analysis indicates that family SES positively conditions the association between loneliness and perceived caregiving burden, with a stronger association observed among higher-SES families. This pattern is not immediately intuitive but becomes more interpretable when considering how resources shape expectations and relative deprivation. Higher-SES parents are more engaged with formal healthcare and rehabilitation services and are more likely to pursue intensive or supplementary interventions (Carpenter et al., 2018; Mitchell et al., 2015). In the context of PWS-where curative treatment remains unavailable-such investments may yield limited observable improvement in patients’ condition, creating a mismatch between effort and outcome (Atkins and Padgett, 2024; Duis et al., 2019; Mackay et al., 2022; Usubini et al., 2024; Chevreul et al., 2016; López-Bastida et al., 2016).

This mismatch can generate a sense of frustration that is not reducible to material constraints. Drawing on the literature on relative deprivation, subjective well-being is shaped not only by absolute resources but also by how individuals evaluate their circumstance in relation to relevant reference groups (Callan et al., 2017; Smith and Pettigrew, 2015). Parents embedded in more resources may hold higher expectations for both treatment efficacy and social support, and may engage in comparison along multiple social reference points. On the one hand, they may look downward and observe that, despite substantial investments in medical care and rehabilitation, their child’s condition does not differ markedly from that of children in less advantaged families who receive fewer interventions. On the other hand, upward or lateral comparisons with their immediate social networks may be equally consequential. When surrounded by similarly advantaged peers whose children are unaffected by rare conditions, parents of children with PWS may experience heightened sense of injustice. Third, the parents may also experience affiliative stigma and loss of face related to their child’s congenital condition, concerns that may be more salient in higher SES families, where children’s outcomes are closely tied to family reputation and parental face in Confucianism-influenced cultural contexts (Dayimu et al., 2026; Leng et al., 2024; Xu, 2023).

At the same time, greater access to medical information does not necessarily alleviate uncertainty; in some cases, it may reinforce awareness of the limits of existing treatments. This informational exposure, combined with potential stigma within high-status social networks, may further intensify feelings of isolation. Taken together, these dynamics help explain why loneliness is more strongly associated with caregiving burden among higher-SES parents, highlighting that psychosocial processes linked to social comparison and expectation can amplify, rather than mitigate, the experiences of caregiving burden in relatively advantaged groups within rare diseases.

## 5 Strengths

To the best of our knowledge, this study provides the first empirical investigation of the relationship between loneliness and caregiving burden among parents of children with PWS in China. It advances our understanding of the complicated interplay between caregiving burden, loneliness, family functioning, and family SES. By introducing moderation analysis, we offered novel and nuanced understanding about family functioning and family SES in the relationship between loneliness and caregiving burden within the resource limited population. From a theoretical perspective, our findings highlight the importance of examining family dynamics when evaluating the negative effect of feelings of loneliness on caregiving burden. Particularly, this study highlights the perceived disadvantageousness of socioeconomically advantaged populations within PWS. In addition, our study draws on a relatively large sample for this population, all participants were recruited within a single country and focused on one rare disease, enhancing the internal consistency and contextual specificity of the findings.

## 6 Limitations

Several limitations of this study should be acknowledged. First, the use of convenience sampling through the “Prader-Willi Syndrome Care and Support Center” may introduce selection bias. Families connected to such organizations may systematically differ from those who are not – they may have greater access to information, higher socioeconomic status, and stronger baseline support. These characteristics could themselves buffer against adverse outcomes, potentially underestimating both the challenges faced by and the effects observed in more isolated or resource-limited families.

Second, the cross-sectional design prevents causal inferences about the relationships between loneliness, family SES, family functioning, and caregiving burden. Although we tested moderation models, these findings should be interpreted as identifying potential explanatory mechanisms rather than establishing causal pathways; the directionality of these associations remains unclear. Longitudinal or experimental designs are needed to establish temporal precedence and to assess how these relationships unfold over time.

Third, this study relied on self-report measures collected at a single time point, raising concerns about common method bias and social desirability. Additionally, our models did not account for several potentially important confounders, including child symptoms related to PWS (e.g., hypotonia), objective indicators of family burden (e.g., expenditure ratios), familial ecology (e.g., relations with extended family), caregiving logistics (e.g., daily time spent on care), and measurement of relative deprivation. Their omission limits our ability to rule out alternative explanations for the observed associations. Future studies employing multi-method, multi-informant designs would help address these methodological constraints.

Finally, while this study provides quantitative evidence on parental caregiving burden and its contributing factors, it does not capture the lived experiences underlying these associations. Qualitative insights into how loneliness, family SES, and family functioning embodied in parents’ daily lives, particularly within the Chinese cultural context of caring for a child with PWS remain absent. Such narratives would deepen understanding of the processes identified here and illuminate culturally nuanced dynamics. Future research employing in-depth qualitative or mixed-method designs would complement these findings and enrich the evidence base for improving family functioning and reducing loneliness.

## 7 Implications

Despite these limitations, this study offers several implications for supporting families of children with PWS in China. First, the findings highlight the importance of strengthening community-based support systems that address parental loneliness and promote family functioning. Programs delivered through community workers and social workers could facilitate social inclusion and provide accessible psychosocial resources for parent caregivers. Second, given the substantial costs associated with long-term care, rehabilitation, and special medical needs, expanded financial assistance and targeted social welfare policies may help alleviate the economic strain faced by families. Finally, the results suggest that loneliness and caregiving burden is not limited to socioeconomically disadvantaged parents. Parents with relatively higher socioeconomic status may also experience higher levels of loneliness which in turn may cause severe caregiving burden, potentially due to social comparison processes and perceived relative deprivation within their reference groups. Interventions that acknowledge these psychosocial dynamics and foster meaningful social connections may help reduce caregiver burden and improve overall family well-being among the parents of children with PWS in mainland China.

## Data Availability

The raw data supporting the conclusions of this article will be made available by the authors, without undue reservation.
