# Correction: Loneliness and parental caregiving burden in families of children with Prader-Willi syndrome in China: the moderating effects of family functioning and socioeconomic status

**DOI:** 10.3389/fpsyg.2026.1956630

**Published:** 2026-08-14

**Authors:** Nueraili Dayimu, Lingli Leng, Gulisitan Kuerbanniyazi, Xiaojing Lin

**Affiliations:** 1 Department of Sociology, Zhejiang University, Hangzhou, China; 2 School of Statistics and Data Science, Xinjiang University of Finance and Economics, Urumqi, China; 3 Prader-Willi Syndrome Care and Support Center of China, Hangzhou, China

**Keywords:** caregiving burden, family function, family SES, loneliness, Prader-Willi syndrome, primary caregiver, rare disease

Affiliation [School of Public Administration, Xinjiang University of Finance and Economics, Urumqi, China] was erroneously included in the paper for author(s) [Gulisitan Kuerbanniyazi]. This affiliation has now been corrected to [School of Statistics and Data Science, Xinjiang University of Finance and Economics, Urumqi, China].

The original version of this article has been updated.

