# Supplementary material for: Loneliness and parental caregiving burden in families of children with Prader-Willi syndrome in China: the moderating effects of family functioning and socioeconomic status
Source: Front Psychol. 2026 May 29;17:1771064. doi: 10.3389/fpsyg.2026.1771064 (PMC13261814; doi:10.3389/fpsyg.2026.1771064)
Supplement: Supplementary file 1 [file Supplementary_file_1.docx]

**Supplementary Figures**


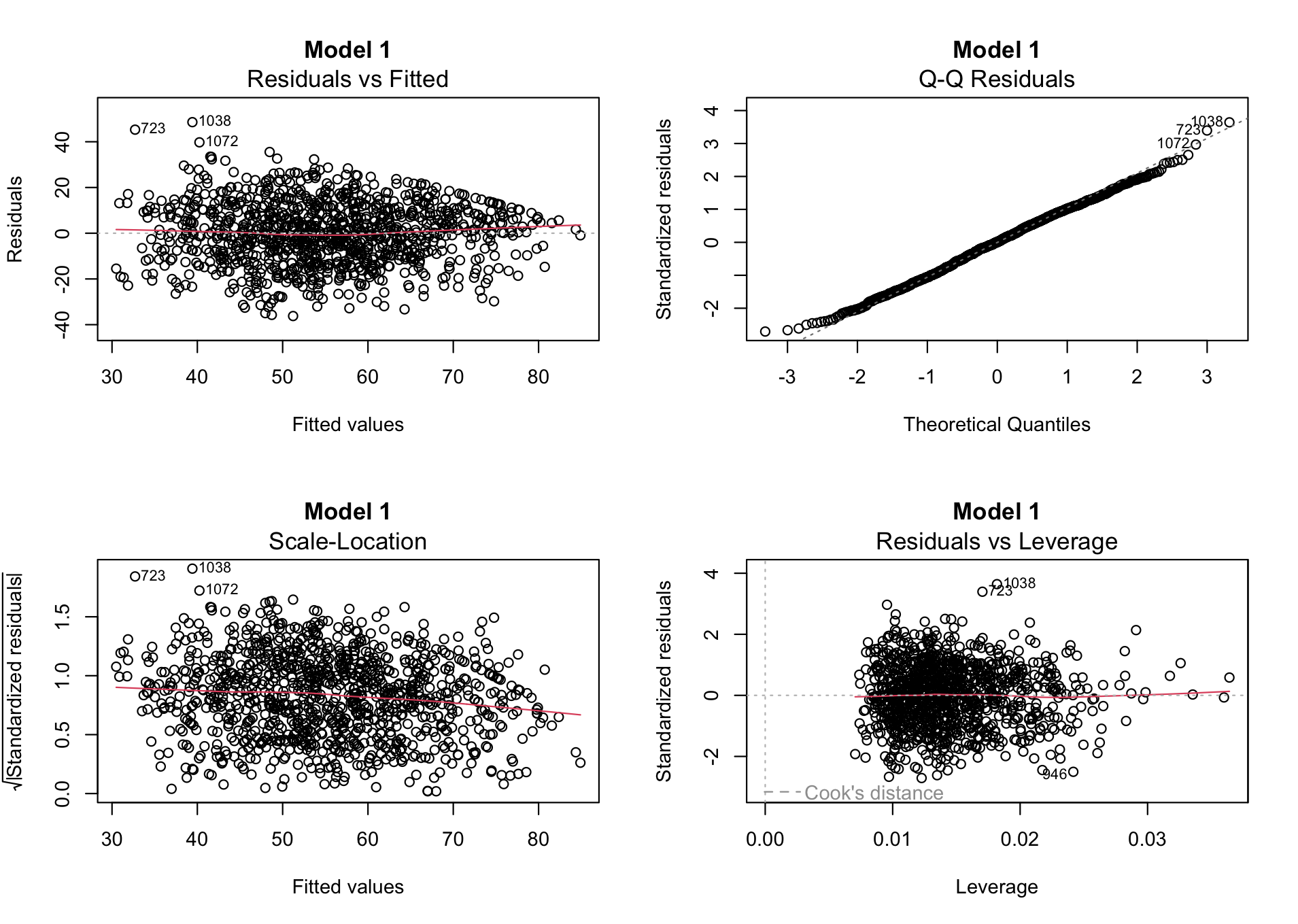


Figure S1. Model assumptions check for model 1


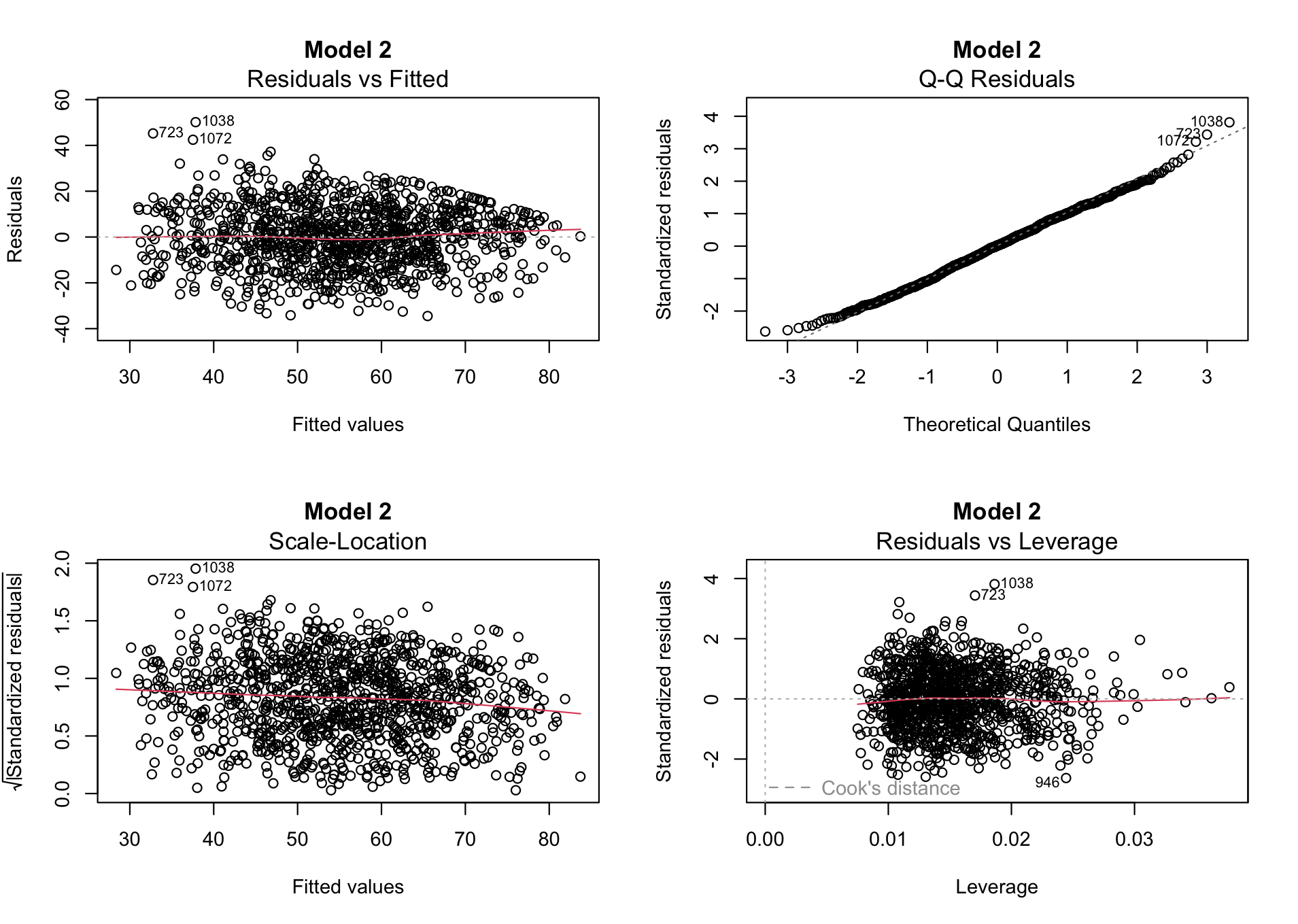


Figure S2. Model assumptions check for model 2


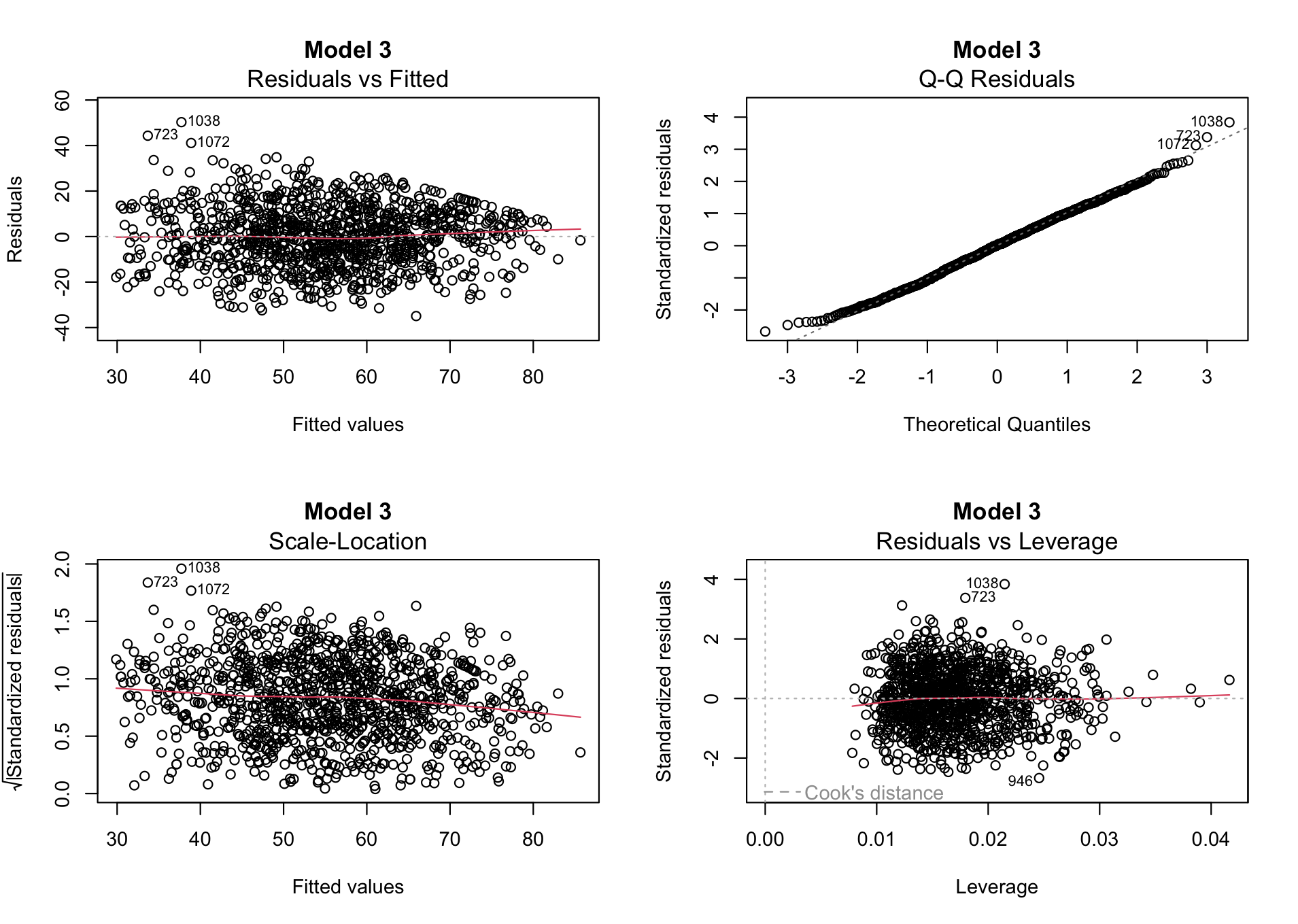


Figure S3. Model assumptions check for model 3


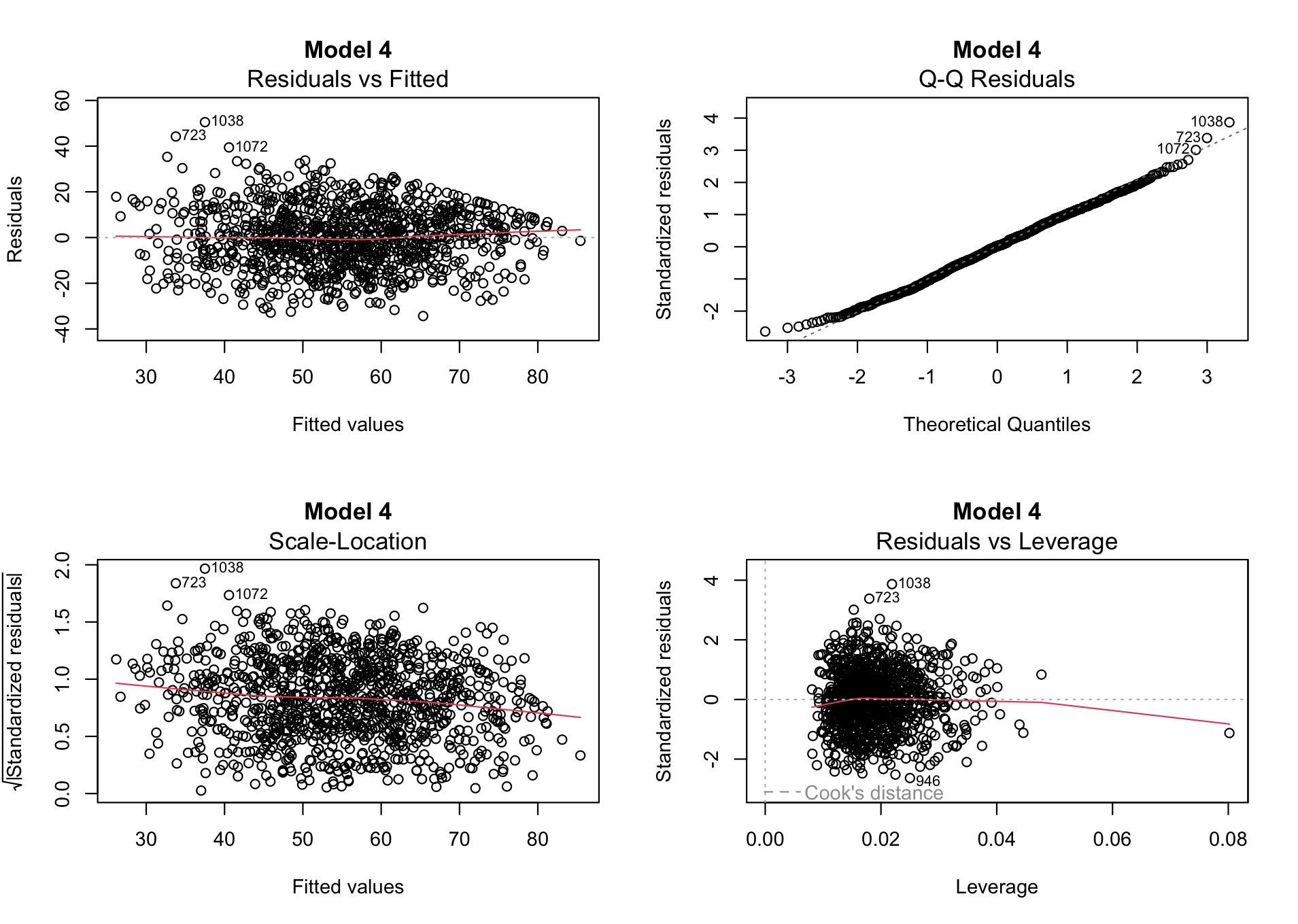


Figure S4. Model assumptions check for model 4 (Moderation model)
